# Quality of sleep and anxiety are related to circadian preference in university students

**DOI:** 10.1371/journal.pone.0238514

**Published:** 2020-09-02

**Authors:** Victor Menezes Silva, Joedyson Emmanuel de Macedo Magalhaes, Leandro Lourenção Duarte

**Affiliations:** Laboratório de Estudos em Neurociências, Cronobiologia & Sono (LENTES), Universidade Federal do Recôncavo da Bahia, Brazil; Technion Israel Institute of Technology, ISRAEL

## Abstract

Chronotype is a circadian phenotype expressed in the preference of individuals to perform their activities and sleep in specific phases along the day. The objective of the study was to identify anxiety levels, quality of sleep and different chronotypes of university students and investigate their possible relationships. This is a cross-sectional study with a quantitative approach, in which 103 undergraduate students answered the Morningness-Eveningness Questionnaire (MEQ) the State and Trait Anxiety Inventory (STAI) and the Pittsburgh Sleep Quality Index (PSQI). There is a relationship between chronotype, quality of sleep and anxiety in the investigated population. Evening chronotype students showed higher anxiety status and have poor sleep quality when compared with morning chronotype students. The high occurrence of anxiety levels and poor sleep quality in evening students may be a consequence of high academic demand in a shift incompatible with the phase delay of the circadian timing system of these individuals.

## Introduction

Living organisms experience several functional changes throughout one day. Examples of such changes are variations in hormone secretion, body temperature, and cognitive performance, among others. Most biological systems present some endogenous circadian timing system that are synchronized by exogenous photic and nonphotic cycles [1].

In humans, the sleep-wake cycle is regulated by the two process-model, proposed by Borbely [2]. The model’s two components consist of a sleep-wake pressure (homeostatic) system, for which a neuroanatomical locus still remains unknown and a circadian timing system, the brain center of which has been localized to the suprachiasmatic nucleus of the hypothalamus, a set of neurons located just above the optic chiasm [3]. This set of neurons forms a network of approximately twenty thousand cells, which receives information from the cells of the retina through the retino-hypothalamic tract [4]. In this perspective, the light-dark cycle is understood as an exogenous synchronizer influencing the endogenous circadian rhythms through specialized photo transducers [5]. There are other synchronizing agents besides the light/dark cycle which also influences the circadian rhythms in humans, for example work and study routines [6] and physical exercise [7].

However, the balance between the circadian system and the synchronizers can be compromised when some exogenous synchronizer undergoes abrupt change. In a historical perspective, an important factor that influences the photoperiod and directly affecting the sleep-wake cycle was the advent of artificial nighttime lighting [8]. Publications that investigate circadian rhythm dysregulation from a mental health perspective have gradually increased over the last few years, especially investigations involving mood, cognition and anxiety disorders [9].

The human organism has a functional characteristic in relation to the different phase allocations of the circadian rhythms, the chronotype. Chronotypes can be divided into categories ranging from a continuum between the morning-type to evening-type individuals. The morning-type wakes up earlier with greater amplitude of disposition, which preferably starts the sleep episode at an earlier time and expresses a decrease in the alert state during the day. The evening-type presents an inversely proportional behavior to the morning-type, therefore, he is preferably awakened in later hours with a smaller amplitude of disposition, with a gradual increase in the alert level during the day. In addition, the individual of the intermediate chronotype is the one who presents, in relation to the phase of their biological rhythms, intermediate position when compared to the individuals of the morning and evening chronotype [10]. The chronotype is a characteristic of the circadian timing system that varies throughout ontogenesis, presents differences between genders and suffers geographical influence from latitude, therefore it should not be considered fixed throughout life [11,12].

University students form a subset of the world's population that appears to be more vulnerable to poor sleep quality and sleep deprivation due to the exhaustive routine of studies and extracurricular activities. Some studies are performed with this specific group, since the relationship between sleep quality and mental health is already established, and poor sleep quality may be a factor of mental stress [13–15]. In the university student setting, some circadian rhythms are misaligned in relation to the non-university population [16]. This is justified by several social determinants of student life: irregular work/rest schedules, worry about examinations, relationships with classmates, the dormitory environment, late bedtimes due to late night internet surfing, excessive vigilance for study, party, work, consumption and / or abuse of alcohol and other drugs, independent living, time management, self-regulation, and living in proximity with peers [15,17–19]. This may be a risk factor for several pathological manifestations of gastrointestinal tract, sleep, anxiety and low levels of attention [20].

Anxiety is a personal and emotional experience depending on the prediction of future circumstances or in the presence of situations determined as dangerous or unpleasant for the individual. During the academic course, the student is constantly confronted with different situations causing psychological pressures and anxiety, such as long hours of study, exhaustive stages, and presentation of seminars and weeks of tests [21]. University students tend to suffer from anxiety symptoms, and there may be a two-way correlation between anxiety and sleep quality [22,23]. The acute fluctuation of mood in the face of momentary situations are characteristic of the anxious state, presenting through sensations such as tension, apprehension, nervousness and worry, while the anxiety trait refers to relatively stable individual differences in behavioral responses [24].

The trait of anxiety is a factor that influences the temporal expression of the sleep-wake cycle, and that the irregularity of the sleep-wake cycle due to school schedules and academic demands seems to contribute to increase the state of anxiety. Thus, it is suggested that the anxiety trait as a endogenous factor that influences the temporal expression of the sleep-wake cycle, and that the irregularity of the sleep-wake cycle due to school hours and academic demands (exogenous factors) seems to contribute to increasing the state of anxiety [25]. The importance of studying the quality of sleep and anxiety of university students lies in the close relationship of these variables with individual and collective health. High levels of anxiety have a negative impact on attention, memory and problem solving (as components of cognitive performance), whereas low and moderate anxiety levels are related to better cognitive performance [26]. Anxiety has been described as one of the most important consequences of sleep deprivation [27] and sleep deprivation has a substantial effect on mood and motor and cognitive performance in humans. [28]. More recently, some authors indicate that active memory consolidation is specifically established during sleep, not only in the neurobehavioral domain, but also in the formation of long-term immune memories [29]. Analysis of the relationship between sleep disorder and academic performance indicates a significant relationship between abnormal daytime sleepiness, total sleeping hours, and academic performance. [14]. Therefore, monitoring anxiety and maintaining quality sleep are crucial for optimal physical, mental and emotional health of students.

Recent studies suggest that chronotype may also be an important marker of the origin of chronic primary insomnia, a risk factor for anxiety disorders [30]. It is also possible to point out a relationship between morningness and performance in high school students [31]. Evening chronotype is associated with sleep disturbances, including shorter sleep duration, poorer sleep quality and daytime sleepiness [32]. These individuals present higher levels of social jet lag, defined by Wittmann et al. (2006) [33] as the absolute difference between mid-sleep time on workdays and mid-sleep time on free days. The social jetlag is associated with several health problems, such as depressed mood, worse academic performance in high school and higher education, obesity and cardio-metabolic risk [34–37]. Evening individuals who have greater irregularity of the sleep-wake cycle may present higher levels of state of anxiety. We asked if the levels of anxiety and quality of sleep in the university students vary according to their chronotype. Thus, the objective of the present study was to identify anxiety levels, quality of sleep and different chronotypes of university students and investigate their possible relationships.

## Materials and methods

### Sample

It is a cross-sectional study with a quantitative approach. The research was carried out at the Centro de Ciências da Saúde of the Universidade Federal do Recôncavo da Bahia, in the city of Santo Antônio de Jesus, Bahia, Brazil. The classes shift is daytime with the start of activities at 7am and ending at 6pm, not having any night classes. The sample consisted of 103 volunteers. A total of 96 students participated in the study, of which 57 were females (59%) with mean age of 22 years (± 2.8) and 39 males (41%) with mean age of 23.15 years (± 4.9). Seven questionnaires were removed from the analysis because they were not correctly answered. The sample profile (majority, young, unmarried and not working) is similar to other studies with university students. The criteria for selection of the sample were to be a student of the courses of the Centro de Ciências da Saúde, to have age equal to or above 18 and below 25 years and to have no history of diagnosis of psychiatric diseases and do not use controlled medications.

### Instruments of data collection

The volunteers completed three questionnaires at the same time: Morningness-Eveningness Questionnaire (MEQ), State-Trait Anxiety Inventory (STAI) and The Pittsburgh Sleep Quality Index (PSQI). All instruments have good psychometric properties, are widely used in scientific literature and have been validated with diverse populations in different countries.

We used the Morningness-Eveningness Questionnaire (MEQ), which was created by Horne and Östberg (1976) [38], translated into Portuguese [39]. The questionnaire consists of 19 multiple choice questions that refer to different everyday situations and individuals declare their preference of the time in the accomplishment of the proposed activities. The result is a numerical value that can vary between 16 and 86 points. Higher MEQ scores represent more morning-oriented preferences for the individual. We categorized the subjects into 3 groups according to the Horne & Östberg score scale: morning-types (n = 28), intermediate-types (n = 40), and evening-types (n = 28).

State-Trait Anxiety Inventory (STAI) is used to measure anxiety scales and was developed by Spielberger et al. [40], translated into Portuguese and validated by Biaggio and Natalício [41] and Gorenstein and Andrade [42]. This is a self-administered questionnaire that has two different scales elaborated to measure two concepts of anxiety: the anxious state (STAI-S), which represents transient state data; and the anxious trait (STAI -T), related to personality data of the individual. Each scale has 20 affirmations for which the volunteers indicate the intensity at that time (STAI—E) and the frequency with which they occur (STAI—T) through a scale of 1 to 4 points. The result is a numerical value and the total score of each scale ranges from 20 to 80 points [43]. The results of the scores were classified according to Mayer et al. (2016) [44] in three types of anxiety levels: low anxiety (< 33 points), moderate anxiety (33 to 49 points), and high anxiety (> 49 points).

The Pittsburgh Sleep Quality Index (PSQI) is a self-administered tool used to evaluate sleep quality and possible sleep disorders in the previous month. It was developed by Buysse et al. in 1989 [45] and validated in Brazil, in the adult population, by Bertolazi et al. in 2011 [46]. The Pittsburgh Sleep Quality Index has score ranges from 0–21 with an abnormal threshold above 5 points.

Before starting to collect the data, the project of this study was sent to the Ethics and Research Committee of the Universidade Federal do Recôncavo da Bahia for evaluation, and it was approved. All subjects included in the study signed a Free and Informed Consent Term authorizing their participation in the research. Students were approached during class breaks, received all the information related to the research and reported if they were interested in to participate. The researchers remained on site to resolve any doubts and wait for the questionnaires to be returned.

### Statistical treatment

For statistical analysis, SPSS software version 23.0 (Statistical Package for the Social Sciences, IBM Corporation, Armonk) was used. For the analysis of the correlation between scores of the MEQ, PSQI and STAI, Pearson's linear correlation coefficient was used. The Kolmogorov-Smirnoff was used to analyze the distribution of data. ANOVA with Tukey´s post-test was used to compare the means of scores between state and trait anxiety groups and the different chronotype profiles. The level of statistical significance of p <0.05 was adopted in all tests.

## Results

**Fig 1:**
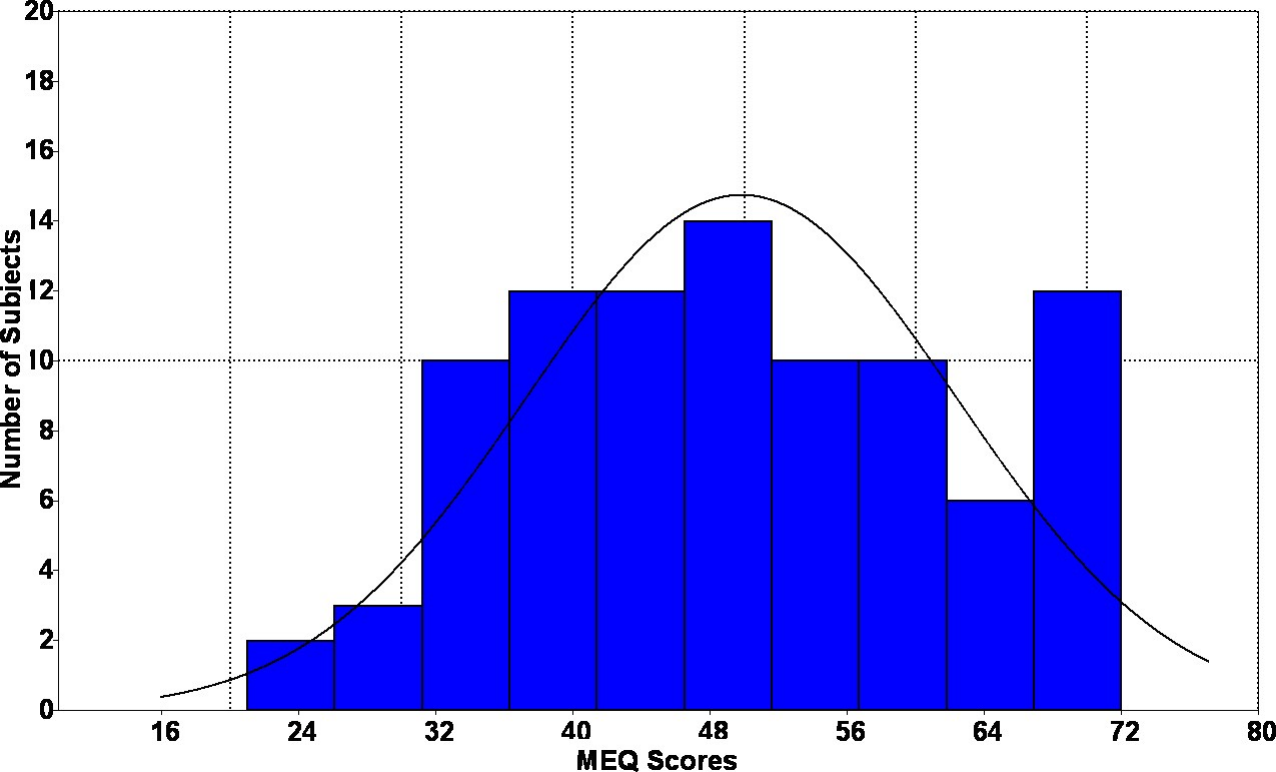
Histogram of the MEQ scores distribution in the study population.

There was homogeneity in the variances (p <0.05) of all analyzed data. From the total sample, the mean value of the Chronotype Questionnaire score was 49.9 points (± 12.4), ranging from 21 to 72 points, and a median of 49.5 points, with normal distribution (Fig 1).

**Fig 2:**
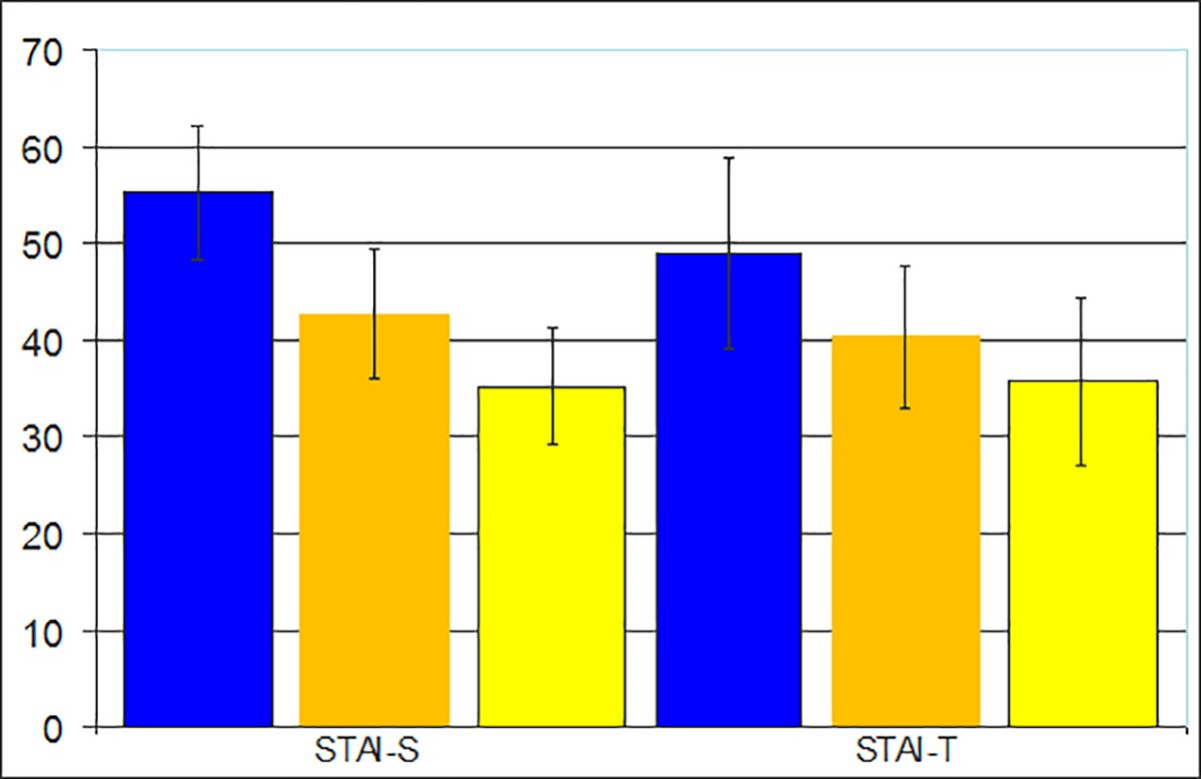
STAI-S and STAI-T means values of the evening-types (blue), intermediated-type (orange) and morning-types (yellow).

**Fig 3:**
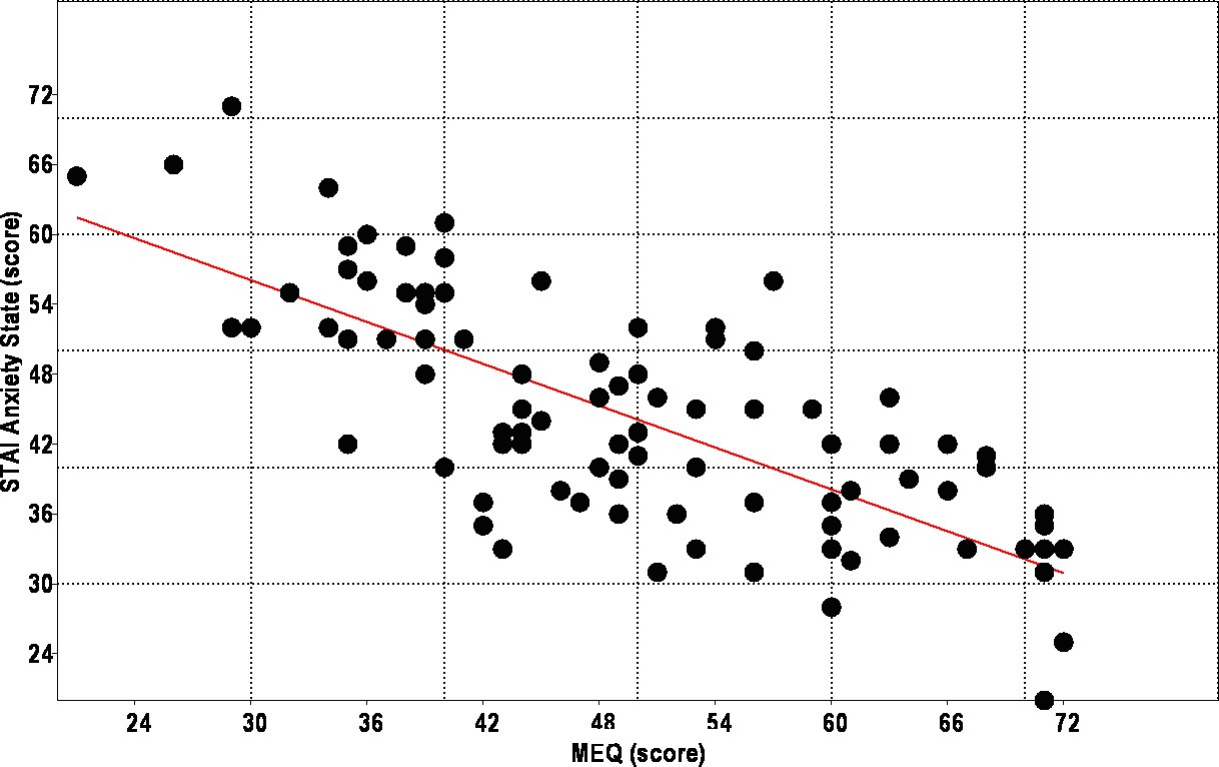
Linear regression analysis between MEQ scores and state anxiety index (STAI-S), (r = -0.743; p = 0.0001).

**Fig 4:**
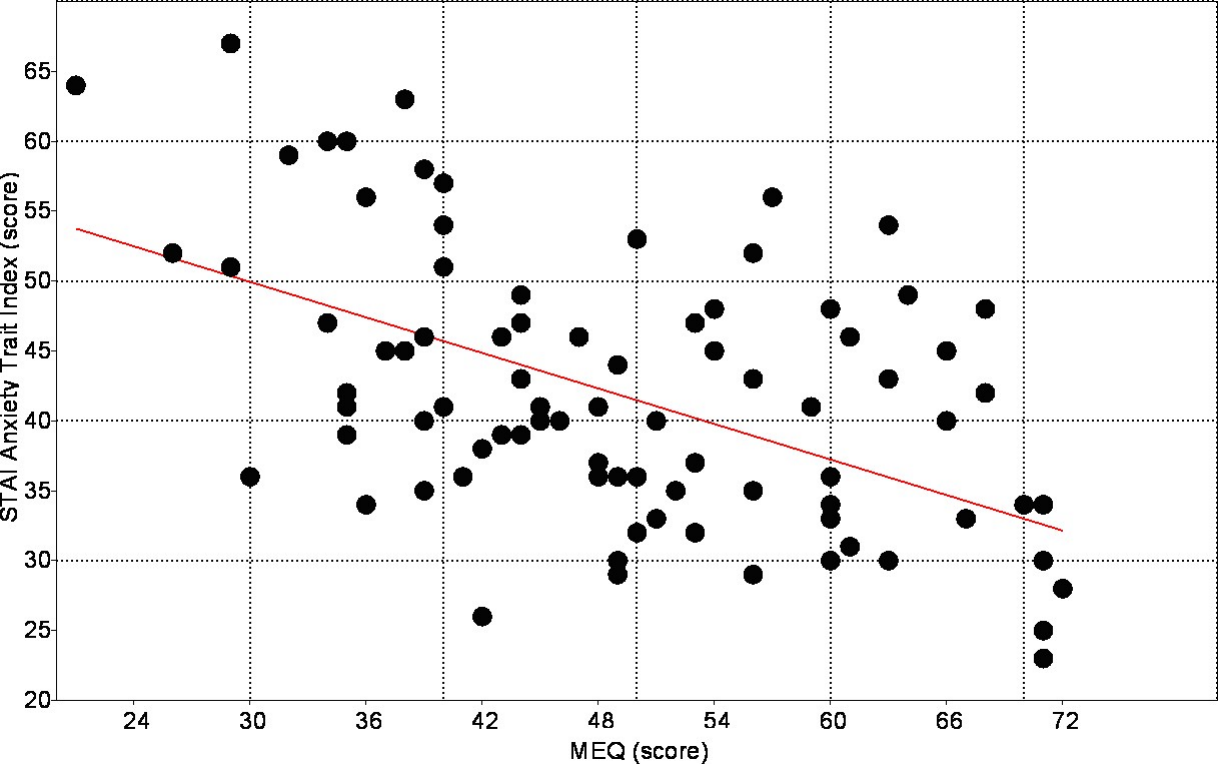
Linear regression analysis between MEQ scores and trace anxiety index (STAI-T), (r = -0.5344; p = 0.0001).

The mean STAI scores were 44.1 (± 10.1) for the anxiety state and 41.5 (± 9.9) for the anxiety trait. No statistically significant difference was found when the sex-separated data were analyzed. A mean of 35.3 (± 6.0) for anxiety state and 35.8 (± 8.7) for anxiety trait was identified in the morning subjects, while in the evening subjects, an average of the anxiety state was 55.2 (± 6.8) and 48.9 (± 9.9) for anxiety trait. The intermediate chronotype subjects showed an average of the 55.2 (± 6.8) for anxiety state and 35.8 (± 8.7) for anxiety trait. Comparing the scores of the STAI categories between the chronotypes, there were higher levels of anxiety in the evening-type as well as lower levels of anxiety in the morning-type in relation to both states and trait of the anxiety (ANOVA, F = 28.6 p < .001). Only in the evening group, the mean anxiety state scores were higher than the anxiety trait scores. In this way, it is possible to affirm that the evening-types students are more anxious in relation to your personality traits (Fig 2). There was a statistically significant negative correlation between STAI-S and MEQ scores (rho = -0.743, p = 0.0001) and between STAI-T and MEQ scores (rho = -0.534, p = 0.0001), revealing that the lower the chronotype score, the higher the anxiety levels (Fig 3, Fig 4).

The mean PSQI score was 6.11 ± 2.44, with 72% of the subjects presenting poor sleep quality, noting that 5.4% of subjects had a score greater than or equal to 10, suggesting a possible sleep disorder. The analysis of the PSQI components showed a correlation between the usual bedtime (rho = 0.291, p <0.005), sleep latency (rho = 0.462, p <0.001) and total sleep time 0.533, p <0.001) with poor sleep quality. A negative correlation was observed between the MEQ and the PSQI scores, and between the MEQ and the STAI (state and trait) scores, while between the PSQI and the STAI (state and trait) scores, positive correlations were found, as described in Table 1. These correlations suggest that the evening type is associated with poor sleep quality, as well as high states of anxiety and anxious personality.

**Table 1:** Rho Pearson coefficients and corresponding p-values of PSQI vs. STAI-S, PSQI vs. STAI-T, PSQI vs. MEQ, MEQ vs. STAI-S, MEQ vs. STAI-T.

	STAI-S	STAI-T	MEQ
PSQI	0.401 (0.001)	0.256 (0.013)	-0.510 (0.001)
MEQ	-0.743 (0.0001)	-0.534 (0.0001)	---------------------

PSQI: Pittsburgh Sleep Quality Index; STAI-S: Trait-State Anxiety Inventory (State); STAI-T: Trait-State Anxiety Inventory (Trait); MEQ: Morningness-Eveningness Questionnaire.

## Discussion

In the present study, the evening chronotype was associated with higher trait and anxiety states and worse sleep quality. Previous studies have shown results in the same direction regarding the relationship between chronotypes and sleep quality [47–52] and anxiety state/trait [30]. Passos and collaborators (2017) reported an association between chronotypes and anxiety in a population of patients with primary chronic insomnia. Thus, our work is the first to find a correlation between evening individuals and anxiety in a non-clinical population.

Almondes and Araújo (2003) [25] used the STAI to evaluate the index of trait and anxiety state in students of the Universidade Federal do Rio Grande do Norte, Brazil (UFRN) who were submitted to the time schedule of classes beginning at 10 am. These authors showed an average of 37.9 (± 9.2) for the anxiety state score and 38.8 (± 10.6) for the anxiety trait, lower values when compared to the sample of the present study. As the daytime classes of the students in our study start at 7am, it is possible that this phase difference is related to the occurrence of a higher level of anxious state in the students compared with those of the Rio Grande do Norte study.

The high occurrence of anxiety levels in evening students may be a consequence of high academic demand in a shift incompatible with the phase delay of the circadian timing system of these individuals. The alert levels of evening individuals are approximately three times higher at 10h than at 7h [53]. With class schedules starting at 7am, the evening students in the present study may show masking of the biological rhythms to be synchronized to the social study cycle. It is possible that this misalignment between endogenous rhythm and social cycle may be related to the occurrence of a higher level of anxious state in the evening students. One study among university students found that nearly 90% had delayed bedtimes with early morning waketimes, resulting in partial sleep deprivation on workdays [54]. In late chronotypes, the constraints of early work schedules lead to an increasing sleep debt over the week that is compensated for on weekends, condition known as social jet lag [55].

One possibility for suggested intervention to reduce the negative impacts of the morning routine on evening students is the delay in the beginning of the classes. However, variations in social routines also imply a change in the pattern of exposure to light, and the consequences of this change are not known [56]. In the United Kingdom, considering the circadian preference patterns of the adolescents, the possibility of official change in the start time of the classes is discussed, to start between 9:30 and 10:00, which currently start between 8:30 and 9:00h [45]. Minges and Redeker (2016) point out that some studies have shown reduced daytime sleepiness, depression, caffeine use, tardiness to class, and trouble staying awake in students submitted to a delay of 25–60 minutes at the beginning of classes. [57]. The Seattle School District delayed the secondary school start time by 55 minutes and there was an increase in average daily sleep duration of 34 min associated with a 4.5% increase in students' median grades and an improvement in attendance [58]. Previous findings suggest that chronotype is related to the performance of cognitive functions in students, that is, individuals show higher mental performance when they do some test, such as theoretical and practical evaluations or seminars, at the time in line to their chronotype [31].

The phenomenon of delayed circadian timing of adolescents is well known in the literature [59,60]. For this reason, studies and interventions in relation to the time daily activities start are aimed at adolescent students. As far as we know, no study with changes in the schedules of university students based on the circadian timing system differences has been carried out until now. We draw attention based on the findings of this study to the need to conduct such studies: modify the start time of the classes in the university collaborates with the reduction of anxiety states of the students? We believe that, at least for 25% of the university population, the adoption of this measure can be beneficial. Regardless of the chronotype, the highest levels of state of anxiety in the population of the present study (mean STAI-S: 44.1) compared with those of Almondes and Araújo (2003) (mean STAI-S: 37.9) can be explained by the different start time of classes, 7:00 in our case and 10:00 in the study mentioned. Although we cannot categorically affirm that this relationship exists, we believe that three hours of difference at the beginning of classes significantly changes the state of anxiety in the university students.

We conclude that evening students are more anxious and have poor sleep quality than morning students. The worse quality of sleep can be explained by the higher sleep latency, shorter total sleep time and late onset of sleep. These characteristics can be justified by academic demand in a shift in which their standard levels of biological rhythms are not compatible, generating successive daily dessynchronizations and collaborating with increase delayed phase of these individuals. The evening-types students are more anxious in relation to your personality traits which indicates a greater irregularity of the sleep-wake cycle than the other chronotypes. Evening students who study in the morning may constitute a vulnerable group for social jet lag, impairment of cognitive and psychological processes, and consequently, worse academic performance and quality of life.

Therefore, it is important to contemplate, as a physiological and individual characteristic, the different tendencies to morningness and eveningness. It is hoped that the results presented here will help to support the different management in academic planning, reinforcing the debate about the need to implement and offer night courses at universities that do not yet offer them, as well as possible reformulations of daytime itineraries, such as example, a delay in the beginning of classes.

Some limitations of the present study can be considered. The cross-sectional design conducted in a relatively small sample of undergraduate students may limit the scope of our conclusions. Proposals for future studies that use more diverse samples, using a longitudinal design, or with the inclusion of objective measures of sleep (wrist actimetry, for example) can make a more robust contribution to the literature. In addition, there is a need to control other factors that contribute to the volunteers' anxiety, including academic rigor, social restrictions, possible substance use, possible changes in the living environment and economic conditions.
